# Roles of Ferredoxin-Dependent Proteins in the Apicoplast of Plasmodium falciparum Parasites

**DOI:** 10.1128/mbio.03023-21

**Published:** 2022-02-15

**Authors:** Russell P. Swift, Krithika Rajaram, Rubayet Elahi, Hans B. Liu, Sean T. Prigge

**Affiliations:** a Department of Molecular Microbiology and Immunology, Johns Hopkins University, Baltimore, Maryland, USA; Albert Einstein College of Medicine; University of Pittsburgh

**Keywords:** apicoplast, iron-sulfur cluster, ferredoxin, ferredoxin reductase, *Plasmodium*, lipoate synthase, Suf, MiaB, IspH, IspG, SufA, Nfu

## Abstract

Ferredoxin (Fd) and ferredoxin-NADP+ reductase (FNR) form a redox system that is hypothesized to play a central role in the maintenance and function of the apicoplast organelle of malaria parasites. The Fd/FNR system provides reducing power to various iron-sulfur cluster (FeS)-dependent proteins in the apicoplast and is believed to help to maintain redox balance in the organelle. While the Fd/FNR system has been pursued as a target for antimalarial drug discovery, Fd, FNR, and the FeS proteins presumably reliant on their reducing power play an unknown role in parasite survival and apicoplast maintenance. To address these questions, we generated genetic deletions of these proteins in a parasite line containing an apicoplast bypass system. Through these deletions, we discovered that Fd, FNR, and certain FeS proteins are essential for parasite survival but found that none are required for apicoplast maintenance. Additionally, we addressed the question of how Fd and its downstream FeS proteins obtain FeS cofactors by deleting the FeS transfer proteins SufA and NfuApi. While individual deletions of these proteins revealed their dispensability, double deletion resulted in synthetic lethality, demonstrating a redundant role in providing FeS clusters to Fd and other essential FeS proteins. Our data support a model in which the reducing power from the Fd/FNR system to certain downstream FeS proteins is essential for the survival of blood-stage malaria parasites but not for organelle maintenance, while other FeS proteins are dispensable for this stage of parasite development.

## INTRODUCTION

One potential source of drug targets in malaria parasites is the essential relict plastid organelle, called the apicoplast. Due to its unique algal evolutionary origin, this organelle is similar to plant chloroplasts and contains biochemical pathways that are highly dissimilar to those of the human host (1–3). The ferredoxin/ferredoxin-NADP^+^ reductase (Fd/FNR) system is one of the few known redox systems within the apicoplast (4–6) and is the only remnant of the photosystem found in plant plastids. The Fd/FNR system provides reducing equivalents to multiple enzymes within the apicoplast (7, 8) and is presumed to be essential for parasite survival. Due to the absence of orthologs in the human host, the Fd/FNR system is an attractive drug target, and several attempts have been made to discover inhibitors (9, 10).

Akin to other nonphotosynthetic plastid Fd/FNR systems, the Plasmodium falciparum FNR reduces the 2Fe-2S-containing Fd through a NADPH-dependent reaction (11–13). The reduced Fd then provides reducing equivalents to other iron-sulfur cluster (FeS) proteins in the apicoplast (7, 8, 14–18). These FeS proteins include lipoic acid synthase (LipA), tRNA-i^6^A37 methylthiotransferase (MiaB), hydroxylmethylbutenyl diphosphate synthase (IspG), and hydroxylmethylbutenyl diphosphate reductase (IspH). LipA is involved in the lipoylation of pyruvate dehydrogenase (PDH) (19), and MiaB is predicted to function as a tRNA-modifying enzyme (20–22). IspG and IspH are involved in the last two steps of the methylerythritol phosphate (MEP) isoprenoid precursor biosynthesis pathway (16–18). All four of these *Plasmodium* proteins, or their orthologs from other systems, have been shown to receive electrons from the Fd/FNR system (14–18).

Since the identification of the Fd/FNR system in the apicoplast of P. falciparum (6), Fd and FNR have been thoroughly characterized using biochemical and structural approaches (11–13, 23–27). In addition to the defined metabolic role of the Fd/FNR system, it has also been suggested that the Fd/FNR redox system has a role in apicoplast maintenance by modulating NADPH balance in the organelle (7, 28, 29). Despite decades of research focused on the Fd/FNR system, including drug development endeavors, there is no definitive evidence that Fd and/or FNR are essential for parasite survival.

It has been previously demonstrated that supplementation with sufficient levels of exogenous isopentenyl pyrophosphate (IPP) allows the disruption and interrogation of essential apicoplast processes (30). Building on this work, our lab engineered a stable P. falciparum parasite line (PfMev) encoding an alternative IPP biosynthesis pathway, which converts exogenously provided mevalonate into the isoprenoid precursors IPP and dimethylallyl pyrophosphate (DMAPP) (31). Proteins essential for apicoplast function can be deleted in the PfMev line; the deletion of proteins responsible for apicoplast maintenance results in loss of the apicoplast organellar genome and localization of nucleus-encoded apicoplast proteins in secretory vesicles rather than in a single intact organelle (31, 32).

In this work, we used the PfMev parasite line to determine the role of the Fd/FNR system in parasite survival and organelle maintenance by deleting both Fd and FNR. Our genetic deletion experiments showed that these proteins are essential for parasite survival. To determine why the Fd/FNR system is responsible for parasite survival, we investigated all four of the FeS proteins that are downstream of Fd, and are thought to rely on Fd for reducing equivalents. Deletion of IspG and IspH revealed that they are essential, suggesting that the electrons provided by the Fd/FNR system to the MEP pathway are required for parasite survival, while LipA and MiaB are dispensable. We searched for additional FeS-containing proteins that may require reducing equivalents from the Fd/FNR system using bioinformatic approaches and were ultimately unsuccessful. To address the role of Fd and all other possible downstream FeS proteins in parasite survival and apicoplast maintenance, we investigated the proteins SufA and NfuApi, which are involved in FeS transfer (7, 33). We show that they are individually dispensable in blood-stage parasites but together are essential for parasite survival, as was shown when they were both deleted. Overall, the work outlined here provides insight into the apicoplast-resident Fd/FNR system and the roles of the proteins upstream and downstream of this redox system.

## RESULTS

### Ferredoxin and ferredoxin NADP+ reductase are essential for parasite survival.

**FIG 1:**
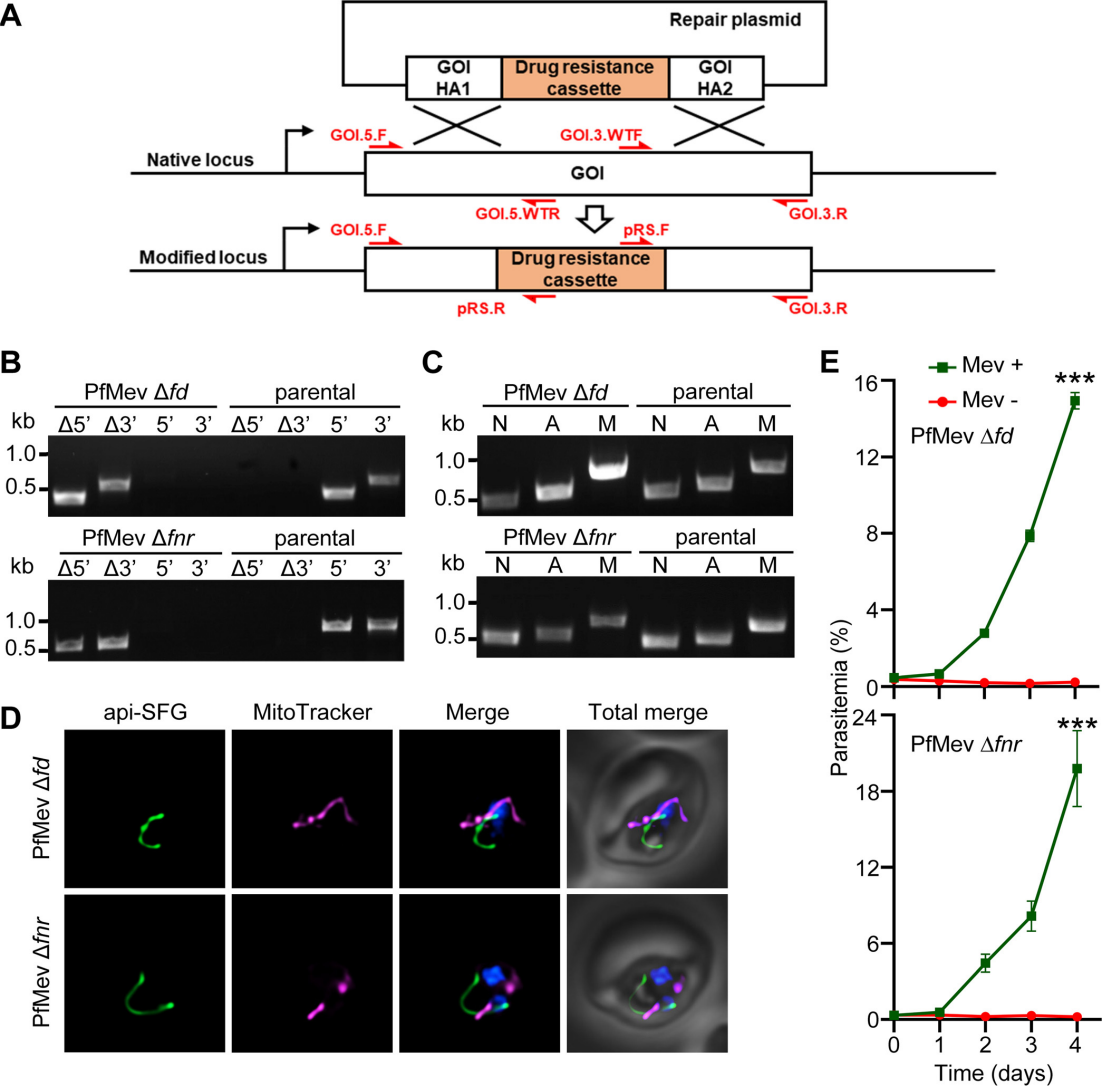
Characterization of PfMev Δ*fd* and PfMev Δ*fnr* parasite lines. (A) General schematic showing the repair plasmid with homology arms (HA) flanking a drug resistance cassette used for gene knockout. The Cas9-endonuclease and guide RNA direct a double-stranded break in the native locus, while the repair plasmid recombines via double-crossover recombination, generating the modified locus. Individual segments are not to scale. The positions and directions of the primers (red font) used for confirmation of the gene knockout are indicated with red arrows. Primer sequences are available in Table S1. GOI, gene of interest. (B) PCR confirming deletion of *fd* or *fnr* in PfMev parasites. The presence of PCR amplicons for the recombinant Δ5′ and Δ3′ loci and lack of amplicons for the endogenous 5′ and 3′ loci in PfMev Δ*fd* (top) and PfMev Δ*fnr* (bottom) parasite lines demonstrate successful gene deletion. The same reactions were also performed in the PfMev line (parental) as a control. Expected amplicon sizes are provided in Table S3. (C) PCR detection of the parasite nuclear (N), apicoplast (A), and mitochondrial (M) genomes by amplifying the *ldh* (N), *sufB* (A), and *cox1* (M) genes from the PfMev Δ*fd* (top) and PfMev Δ*fnr* (bottom) parasite lines, with the PfMev parasite line (parental) serving as a positive control. Successful amplification of *sufB* in the PfMev Δ*fd* and PfMev Δ*fnr* parasites indicates presence of the apicoplast genome. (D) Live epifluorescence microscopy of PfMev Δ*fd* (top) and PfMev Δ*fnr* (bottom) parasites. In both parasite lines, a single intact apicoplast is observed. The api-SFG protein (green; see Materials and Methods for details on api-SFG) marks the apicoplast, MitoTracker (magenta) stains the mitochondrion, and nuclear DNA is stained with DAPI (blue). For both lines, imaging was done in the presence of mevalonate. Each image depicts a field of 10 μm by 10 μm. (E) Growth curve of PfMev Δ*fd* (top) and PfMev Δ*fnr* (bottom) parasites. Asynchronous parasites from each line were cultured in the presence or absence of 50 μM mevalonate (Mev). Parasitemia was determined every 24 h via flow cytometry for 4 days. Data from two independent biological replicates, each with four technical replicates, are shown. Error bars represent standard errors of the means. Two-way analysis of variance (ANOVA; Šidák-Bonferroni method) was used. ***, *P* < 0.001.

Previous work speculated that disruption of the Fd/FNR system may also perturb NADPH homeostasis, resulting in the disruption of the organelle (7, 28, 29). To test whether Fd (gene ID PF3D7_1318100) and/or FNR (gene ID PF3D7_0623200) are essential for blood-stage parasite survival or apicoplast maintenance, we deleted the genes encoding these proteins in the PfMev parasite line using CRISPR-Cas9-mediated genome editing (34) (Fig. 1A) under mevalonate supplementation (31). Successful gene deletions were confirmed by PCR (Fig. 1B). To determine the effect of gene deletions on the apicoplast genome, we attempted to amplify *sufB*, a gene from the apicoplast genome, in both PfMev Δ*fd* and PfMev Δ*fnr* parasite lines. Successful amplification of the *sufB* gene indicated that both lines retained the apicoplast genome (Fig. 1C). Apicoplast morphology was assessed using live epifluorescence microscopy to observe the apicoplast-trafficked Super Folder Green (api-SFG) fluorescent protein (35). An intact apicoplast was seen in both the parasite lines, demonstrating that these deletions did not result in organelle disruption (Fig. 1D). Additionally, we tested the growth of the PfMev Δ*fd* and PfMev Δ*fnr* parasite lines in the presence or absence of mevalonate supplementation and found that both lines are reliant on mevalonate supplementation for growth (Fig. 1E). We also show that both gene deletion parasite lines do not show any significant growth defect compared with the parental PfMev line when grown in the presence of mevalonate (Fig. S1A). Taken together, these results demonstrate that Fd and FNR are essential for blood-stage parasite survival but not for apicoplast maintenance.

10.1128/mBio.03023-21.1 FIG S1 Growth comparison of gene deletion and parental parasite lines. (A) Growth comparison of PfMev Δ*fd*, PfMev Δ*fnr*, and the PfMev parental line in the presence of exogenous mevalonate supplementation. (B) Growth comparison of PfMev Δ*lipA*, PfMev Δ*miaB*, and the PfMev parental line. (C) Growth comparison of PfMev Δ*ispG*, PfMev Δ*ispH*, and the PfMev parental line in the presence of exogenous mevalonate supplementation. (D) Growth comparison of PfMev Δ*sufA*, PfMev Δ*nfuApi,* PfMev Δ*sufA* Δ*nfuApi*, and the PfMev parental line in the presence of exogenous mevalonate supplementation. In all cases, asynchronous parasites were cultured for a complete growth cycle (∼48 h), after which parasitemia was determined. The ratio of the final and initial parasitemia (fold increase) represents the growth rate. Data are from five biological replicates, each with technical duplicates; error bars represent standard deviations. ns, nonsignificant (two-way ANOVA, Mann-Whitney U test); *P* > 0.05. Download FIG S1, TIF file, 0.1 MB. Copyright © 2022 Swift et al. 2022 Swift et al. https://creativecommons.org/licenses/by/4.0/ This content is distributed under the terms of the Creative Commons Attribution 4.0 International license.

10.1128/mBio.03023-21.2 TABLE S1 Primers and oligonucleotide used in this study. Download Table S1, DOCX file, 0.03 MB. Copyright © 2022 Swift et al. 2022 Swift et al. https://creativecommons.org/licenses/by/4.0/ This content is distributed under the terms of the Creative Commons Attribution 4.0 International license.

10.1128/mBio.03023-21.4 TABLE S3 Primer combinations and expected amplicon sizes for genotype validation PCRs. Download Table S3, DOCX file, 0.03 MB. Copyright © 2022 Swift et al. 2022 Swift et al. https://creativecommons.org/licenses/by/4.0/ This content is distributed under the terms of the Creative Commons Attribution 4.0 International license.

### LipA and MiaB are dispensable for blood-stage parasite growth.

**FIG 2:**
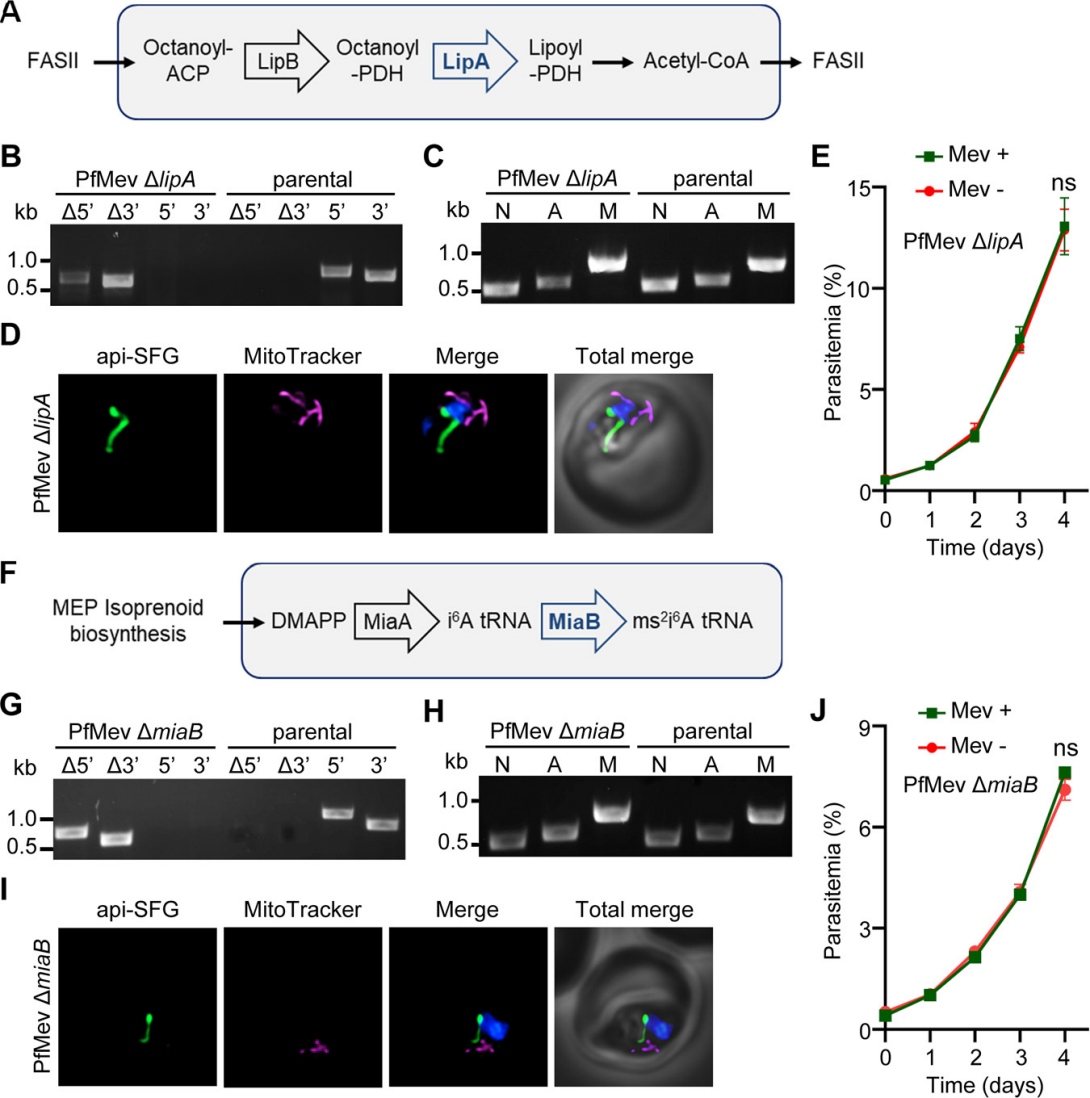
Characterization of PfMev Δ*lipA* and PfMev Δ*miaB* parasite lines. (A) Simplified representation of the apicoplast lipoylation pathway. Lipoylation of the PDH E2 subunit is catalyzed by LipA (in blue), enabling PDH E2 to generate acetyl-CoA for the FASII pathway. LipA uses 4Fe-4S clusters as a cofactor. For simplification, PDH E2 is denoted by PDH in the schematic. FASII, type II fatty acid synthesis; ACP, acyl carrier protein; LipB, lipoate transferase; LipA, lipoate synthase; PDH, pyruvate dehydrogenase. (B) Genotyping PCR confirming *lipA* deletion in the PfMev Δ*lipA* transgenic parasite line. Presence of the Δ5′ and Δ3′ amplicons, but absence of endogenous 5′ and 3′ amplicons, indicates successful gene deletion. The PfMev parasite line (parental) was used as a control. Expected amplicon sizes are provided in Table S3. (C) Parasite nuclear (N), apicoplast (A), and mitochondrial (M) genome detection by PCR amplification of *ldh* (N), *sufB* (A), and *cox1* (M) genes from the PfMev Δ*lipA* and PfMev (parental) parasite lines. Successful amplification of *sufB* in the PfMev Δ*lipA* parasite line indicates retention of the apicoplast genome. (D) Live epifluorescence microscopy of the PfMev Δ*lipA* parasite line, showing a single intact organelle. The api-SFG protein (green) labels the apicoplast, the mitochondrion is stained with MitoTracker (magenta), and nuclear DNA is stained with DAPI (blue). Imaging was done in the absence of exogenous mevalonate. Each image depicts a field of 10 μm by 10 μm. (E) Growth curve of the PfMev Δ*lipA* parasite line. Asynchronous parasites were cultured in the presence or absence of 50 μM mevalonate (Mev) for 4 days. Parasitemia was determined every 24 h by flow cytometry. Data are from two independent biological replicates, each with four technical replicates. Error bars represent standard errors of the means. ns, nonsignificant (two-way ANOVA, Šidák-Bonferroni method); *P* > 0.05. (F) Biosynthesis of the ms^2^i^6^A37 tRNA modification in the apicoplast. This modification at the adenosine (A) 37 position of specific tRNAs is catalyzed by MiaA and MiaB (in blue) using DMAPP from the MEP isoprenoid precursor biosynthesis pathway. DMAPP, dimethylallyl pyrophosphate; MiaA, tRNA-isopentenylpyrophosphate transferase; i^6^A, *N*^6^-isopentenyl adenosine; MiaB, tRNA-i^6^A37 methylthiotransferase; ms^2^i^6^A, 2-methylthiol-*N*^6^-isopentenyl adenosine. (G) Genotyping PCR confirming *miaB* deletion in the PfMev Δ*miaB* parasite line. Presence of the Δ5′ and Δ3′ amplicons indicates successful gene deletion. The PfMev parasite line (parental) was used as a control. Expected amplicon sizes are provided in Table S3. (H) Parasite nuclear (N), apicoplast (A), and mitochondrial (M) genome detection by PCR amplification of *ldh* (N), *sufB* (A), and *cox1* (M) genes from the PfMev Δ*miaB* and PfMev (parental) parasite lines. Successful amplification of *sufB* in the PfMev Δ*miaB* parasite line indicates retention of the apicoplast genome. (I) Live epifluorescence microscopy of the PfMev Δ*miaB* parasite line, showing a single intact organelle. The api-SFG protein (green) labels the apicoplast, the mitochondrion is stained with MitoTracker (magenta), and nuclear DNA is stained with DAPI (blue). These parasites were imaged in the absence of exogenous mevalonate. Each image depicts a field of 10 μm by 10 μm. (J) Growth curve of the PfMev Δ*miaB* parasite line. Asynchronous parasites were cultured in the presence or absence of 50 μM mevalonate (Mev) for 4 days. Parasitemia was determined every 24 h by flow cytometry. Data are from two independent biological replicates, each with four technical replicates. Error bars represent standard errors of the means. ns, nonsignificant (two-way ANOVA, Šidák-Bonferroni method); *P* > 0.05.

We investigated the roles of other apicoplast-localized proteins that are dependent on Fd for reducing power to determine which are responsible for the essential phenotype of the Fd/FNR system. Based on its absorbance spectrum, LipA (gene ID PF3D7_1344600) is a 4Fe-4S protein (7). In Toxoplasma gondii (a close relative of P. falciparum) LipA interacts with P. falciparum Fd in a yeast two-hybrid assay, suggesting that it is able to receive electrons donated by reduced Fd (15). The apicoplast localized LipA is responsible for the lipoylation of the PDH E2 subunit, enabling it to generate acetyl coenzyme A (acetyl-CoA) for use by the FASII pathway (19) (Fig. 2A). While the FASII pathway is dispensable for blood-stage parasites (36), LipA has remained refractory to deletion (37). We were able to delete *lipA* in the PfMev parasite line under continuous supplementation with mevalonate (Fig. 2B). We found that the deletion of LipA had no effect on the apicoplast genome as evidenced by amplification of *sufB* (Fig. 2C), and via live epifluorescence microscopy we confirmed that the apicoplast remained intact (Fig. 2D). We also showed that the PfMev Δ*lipA* parasites can grow without mevalonate supplementation (Fig. 2E) and have no significant growth defect in comparison to the parental PfMev line (Fig. S1B).

Next, we targeted MiaB (gene ID PF3D7_0622200) for deletion. Although P. falciparum MiaB has not been studied, bacterial orthologs are 4Fe-4S proteins that have been shown to receive electrons from bacterial Fd (14, 38). MiaB is believed to function as a tRNA-modifying enzyme, acting as a tRNA methylthiotransferase (20). MiaB activity typically occurs downstream of a prior tRNA modification mediated by MiaA, which acts as an isopentenyltransferase using DMAPP (39) (Fig. 2F). In other systems, these modifications have been shown to be important for proper protein expression levels, nonsense suppression, translational fidelity, and stabilizing base pairing between the codon and anticodon, with deletions resulting in altered rates of translation and differences in gene expression (40–44). To investigate the role and importance of MiaB, we generated PfMev Δ*miaB* parasites (Fig. 2G). These parasites retained the apicoplast genome (Fig. 2H), and the intact apicoplast was confirmed via live epifluorescence microscopy (Fig. 2I). Like PfMev Δ*lipA* parasites, the PfMev Δ*miaB* parasites were also able to grow without mevalonate supplementation (Fig. 2J) and grow at a rate similar to that of the parental line (Fig. S1B). These parasites did not show any delayed-acting phenotype when cultured for a long period of time (data not shown), ruling out the possibility of delayed effect on the translational fidelity. These findings demonstrate that MiaB and LipA are dispensable in blood-stage malaria parasites.

### MEP isoprenoid biosynthesis Fd-dependent proteins are essential for parasite survival.

**FIG 3:**
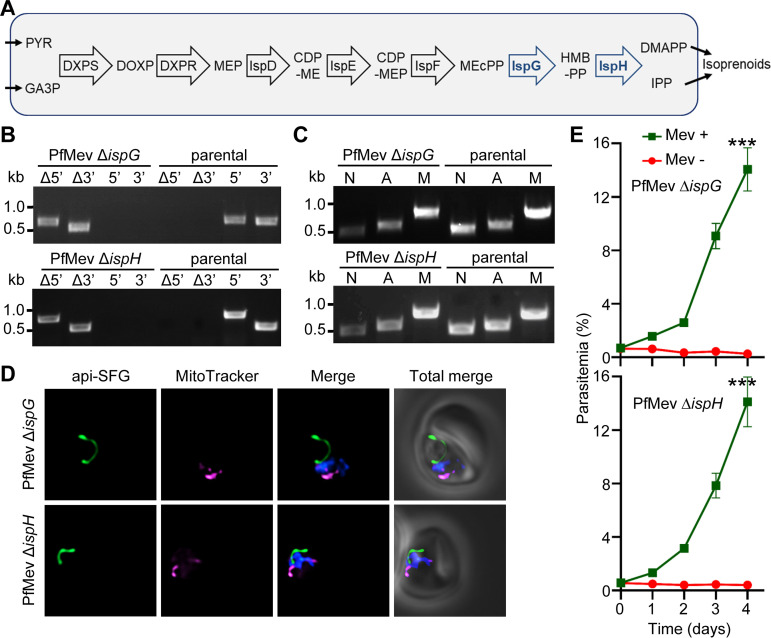
Characterization of PfMev Δ*ispG* and PfMev Δ*ispH* parasite lines. (A) Diagram of the methylerythritol phosphate (MEP) isoprenoid precursor biosynthesis pathway. The last two enzymes (in blue) of the pathway use 4Fe-4S clusters as a cofactor. PYR, pyruvate; GA3P, glyceraldehyde-3-phosphate; DXPS, 1-deoxy-*d*-xylulose 5-phosphate synthase; DOXP, 1-deoxy-*d*-xylulose 5-phosphate; DXPR, DOXP reductoisomerase; MEP, 2-*C*-methylerythritol 4-phosphate; IspD, MEP cytidyltransferase; CDP-ME, 4-diphosphocytidyl-2-*C*-methylerythritol; IspE, CDP-ME kinase; CDP-MEP, 4-diphosphocytidyl-2-*C*-methylerythritol 2-phosphate; MEcPP, 2-*C*-methylerythritol 2,4-cyclodiphosphate; IspF, MEcPP synthase; HMB-PP, (E)-4-hydroxy-3-methyl-but-2-enyl diphosphate; IspG, HMB-PP synthase; IspH, HMB-PP reductase; DMAPP, dimethylallyl pyrophosphate; IPP, isopentenyl pyrophosphate. (B) Genotyping PCR to confirm deletion of *ispG* or *ispH* in PfMev parasites. In both PfMev Δ*ispG* (top) and PfMev Δ*ispH* (bottom) parasite lines, presence of PCR products for the recombinant Δ5′ and Δ3′ loci and lack of amplification for the endogenous 5′ and 3′ loci demonstrate successful gene deletion. The same reactions were also conducted with the PfMev line (parental) as a control. Expected amplicon sizes are provided in Table S3. (C) Parasite nuclear (N), apicoplast (A), and mitochondrial (M) genome detection by PCR amplifying *ldh* (N), *sufB* (A), and *cox1* (M) genes from the PfMev Δ*ispG* (top) and PfMev Δ*ispH* (bottom) parasite lines, with the PfMev parasite line (parental) serving as a positive control. Successful amplification of *sufB* in the PfMev Δ*ispG* and PfMev Δ*ispH* parasites indicates presence of the apicoplast genome. (D) Live epifluorescence microscopy of PfMev Δ*ispG* (top) and PfMev Δ*ispH* (bottom) parasites. In both parasite lines, a single intact apicoplast is seen. The api-SFG protein (green) labels the apicoplast, the mitochondrion is stained with MitoTracker (magenta), and nuclear DNA is stained with DAPI (blue). Imaging was done in the presence of mevalonate for both parasite lines. Each image depicts a field of 10 μm by 10 μm. (E) Growth curve of PfMev Δ*ispG* (top) and PfMev Δ*ispH* (bottom) parasites. Asynchronous parasites from each line were cultured in the presence or absence of 50 μM mevalonate (Mev). Parasitemia was determined every 24 h by flow cytometry for 4 days. Data from two independent biological replicates, each with four technical replicates, are shown. Error bars represent standard errors of the means. ***, *P* < 0.001 (two-way ANOVA, Šidák-Bonferroni method).

The two FeS-dependent MEP isoprenoid precursor biosynthesis pathway enzymes IspG (gene ID PF3D7_1022800) and IspH (gene ID PF3D7_0104400) are thought to be essential, since they are responsible for the last two steps of IPP production (16, 17, 22) (Fig. 3A). Biochemical studies showed that the 4Fe-4S cluster of IspH receives an electron from the Fd/FNR system (16), and the same has been hypothesized for IspG (17). To investigate the roles of IspG and IspH, we deleted the genes encoding these proteins in the PfMev parasite line (Fig. 3B). We found that the apicoplast remains intact in both the PfMev Δ*ispG* and PfMev Δ*ispH* parasites lines, as evidenced by amplification of *sufB* (Fig. 3C), with the phenotype confirmed via live epifluorescence microscopy (Fig. 3D). We also found that both genes are essential for blood-stage parasite survival, since both the PfMev Δ*ispG* and PfMev Δ*ispH* parasite lines failed to replicate upon the removal of mevalonate (Fig. 3E). PfMev Δ*ispG* and PfMev Δ*ispH* parasite lines also grow at a rate similar to that of the parental line when supplemented with mevalonate (Fig. S1C). Taken together, these results show that IspG and IspH are essential for blood-stage parasite survival and are not required for the maintenance of the apicoplast.

### Bioinformatic approaches did not identify additional apicoplast FeS proteins.

The results above show that the reducing equivalents provided by the Fd/FNR systems to the MEP pathway FeS-dependent proteins are necessary for parasite survival and that LipA and MiaB are dispensable. However, there may be other essential FeS proteins which rely on reducing equivalents from the Fd/FNR system but have yet to be identified. To search for additional apicoplast-trafficked FeS-containing proteins, we analyzed the amino acid sequences of every protein encoded by the P. falciparum 3D7 genome through the program MetalPredator to identify predicted FeS-binding proteins (45, 46). We generated a list of all potential FeS-binding proteins (Table 1) and used PlasmoAP to predict the likelihood of apicoplast trafficking (47). We also used data from forward genetic screens in P. falciparum (48) and Plasmodium berghei (49) to predict the essentiality of these genes. This analysis returned many known FeS proteins but did not identify any novel, putatively apicoplast-localized proteins. It is always possible, however, that this kind of analysis can miss some FeS proteins, since there is not one amino acid sequence motif or protein fold that defines these proteins.

**TABLE 1:** Potential iron-sulfur cluster binding proteins in P. falciparum^*a*^

Gene ID (PF3D7_)^*b*^	Gene name	Prediction^*c*^		Essentiality			MitoProbe export probability (81)
		Signal peptide	Transit peptide	P. falciparum (48)	P. berghei (49)	P. falciparum (this study)	
**1344600**	**LipA; Lipoyl synthase**	**++**	**++**	**Essential**	**Dispensable**	**Dispensable**	**0.6838**
**0921400**	**NfuApi; NifU-like scaffold protein**	**++**	**++**	**Essential**	**Dispensable**	**Dispensable**	**0.2084**
1103400	SufD FeS cluster assembly protein	++	++	Essential	Essential		0.5468
0716600	SufS cysteine desulfurase	++	++	Slow/dispensable	Slow		0.9182
**0522700**	**SufA; Iron-sulfur assembly protein**	**++**	**++**	**Essential**	**Dispensable**	**Dispensable**	**0.9862**
**1318100**	**Fd; Ferredoxin**	**++**	**++**	**Slow/dispensable**	**Essential**	**Essential**	**0.8117**
0910800	Cytosolic FeS assembly factor NBP35	++	++	Slow	Essential		0.9760
**0622200**	**MiaB; Radical SAM protein**	**+**	**++**	**Dispensable**	**Dispensable**	**Dispensable**	**0.9952**
**1022800**	**IspG; hydroxylmethylbutenyl diphosphate synthase**	**+**	**++**	**Essential**	**Essential**	**Essential**	**0.8285**
0910900	DNA primase large subunit	−	++	Essential	Essential		0.0509
1342100	Aconitate hydratase	−	++	Slow/dispensable	Essential		0.8460
1214600	Adrenodoxin-type ferredoxin	−	++	Slow/essential	Essential		0.8299
1454500	ISU iron sulfur cluster assembly protein	−	++	Essential	Essential		0.6647
0207200	IscA1 iron-sulfur assembly protein	−	++	Essential	Essential		0.9521
0727200	NFS cysteine desulfurase	−	++	Slow/essential	Essential		0.1566
0720400	Apoptosis-inducing factor	−	++	Dispensable	ND		0.8670
0614800	Endonuclease III homologue	−	++	Dispensable	ND		0.5453
1220500	Ribosome biogenesis protein TSR3	−	++	Essential	ND		0.1141
1361600	FeS assembly protein IscX	−	++	Essential	ND		0.9667
1439400	Cytochrome *b*-*c*_1_ complex subunit Rieske	−	++	Dispensable	ND		0.9782
0927300	Fumarate hydratase	−	++	Essential	ND		0.8939
1212800	Iron-sulfur subunit of succinate dehydrogenase	−	++	Dispensable	Dispensable		0.1521
0709200	Glutaredoxin-like protein	−	++	Dispensable	Dispensable		0.6975
0416700	CDGSH iron-sulfur domain-containing protein	−	++	Dispensable	Dispensable		0.3166
0930900	NifU-like protein	−	++	Dispensable	Dispensable		0.9977
1306300	SAM dependent methyltransferase	−	++	Dispensable	Dispensable		0.9961
API04700	SufB FeS cluster assembly protein	−	++	ND	ND		0.9380
1472700	DNA-directed RNA polymerase, alpha subunit	+	+	Essential	ND		0.4346
1406900	Radical SAM protein	−	+	Essential	Essential		0.1722
0322500	IscA2 iron-sulfur assembly protein	−	+	Dispensable	ND		0.4360
**0104400**	**IspH; hydroxylmethylbutenyl diphosphate reductase**	**−**	**−**	**Dispensable**	**ND**	**Essential**	**0.5404**
1017000	DNA polymerase delta catalytic subunit	−	−	Essential	Essential		0.1900
0934100	TFIIH basal transcription factor complex helicase XPD subunit	−	−	Essential	Essential		0.0106
1128500	FeS assembly protein	−	−	Essential	Essential		0.1301
1227800	Elongator complex protein 3	−	−	Slow/dispensable	Essential		0.0660
1022900	CDGSH iron-sulfur domain-containing protein	−	−	Dispensable	Essential		0.0112
0524900	tRNA-YW synthesizing protein	−	−	Dispensable	Essential		0.0527
1458700	Exonuclease V, mitochondrial	−	−	Essential	Slow		0.0841
1238800	Acyl-CoA synthetase	−	−	Essential	Slow		0.2453
1435300	NAD(P)H-dependent glutamate synthase	−	−	Dispensable	Slow		0.0131
0606900	Glutaredoxin-like protein	−	−	Essential	Slow		0.0063
1408400	FANCJ-like helicase	−	−	Essential	Slow		0.0139
1143300	DNA-directed RNA polymerases I and III subunit RPAC1	−	−	Essential	ND		0.0677
0515800	BolA-like protein	−	−	Essential	ND		0.2887
1415200	DNA-directed RNA polymerases I and III subunit RPAC2	−	−	Essential	ND		0.0476
1413800	Diphthamide biosynthesis protein 1	−	−	Slow/dispensable	ND		0.0176
0923000	DNA-directed RNA polymerase II subunit RPB3	−	−	Essential	ND		0.0248
0614200	Cytosolic FeS assembly factor NAR1	−	−	Essential	ND		0.0141
1324500	DEAD box helicase	−	−	Essential	ND		0.4691
0302700	CDGSH iron-sulfur domain-containing protein	−	−	Essential	ND		0.0176
1129500	A/G-specific adenine glycosylase	−	−	Dispensable	Dispensable		0.0348
0306300	Glutaredoxin 1	−	−	Essential	Dispensable		0.4154
0824600	FeS assembly protein DRE2	−	−	Essential	Essential		0.1138
1368200	ABC transporter E family member 1	−	0	Essential	Essential		0.0535

a Proteins investigated in this work are in bold. ND, not determined.

b Gene IDs are from PlasmoDB (46).

c Signal peptide and apicoplast transit peptide predictions were made using PlasmoAP (47) as implemented in PlasmoDB (++, very likely; +, likely; 0, undecided; − unlikely).

### FeS transfer proteins are functionally redundant.

The Fd/FNR system was originally hypothesized to be essential for maintaining the apicoplast (7); however, we have shown that the organelle remains intact after deletion of Fd, FNR, or other downstream FeS-dependent proteins. As noted above, it is possible that there are additional unidentified apicoplast-localized FeS proteins, and these proteins could have a role in apicoplast maintenance. We hypothesized that if we interfered with the transfer of FeS cofactors assembled by the apicoplast SUF (sulfur utilization factor) FeS biosynthesis pathway (7, 33), we would be able to simultaneously inactivate all the known (and unknown) FeS-dependent proteins, including Fd.

**FIG 4:**
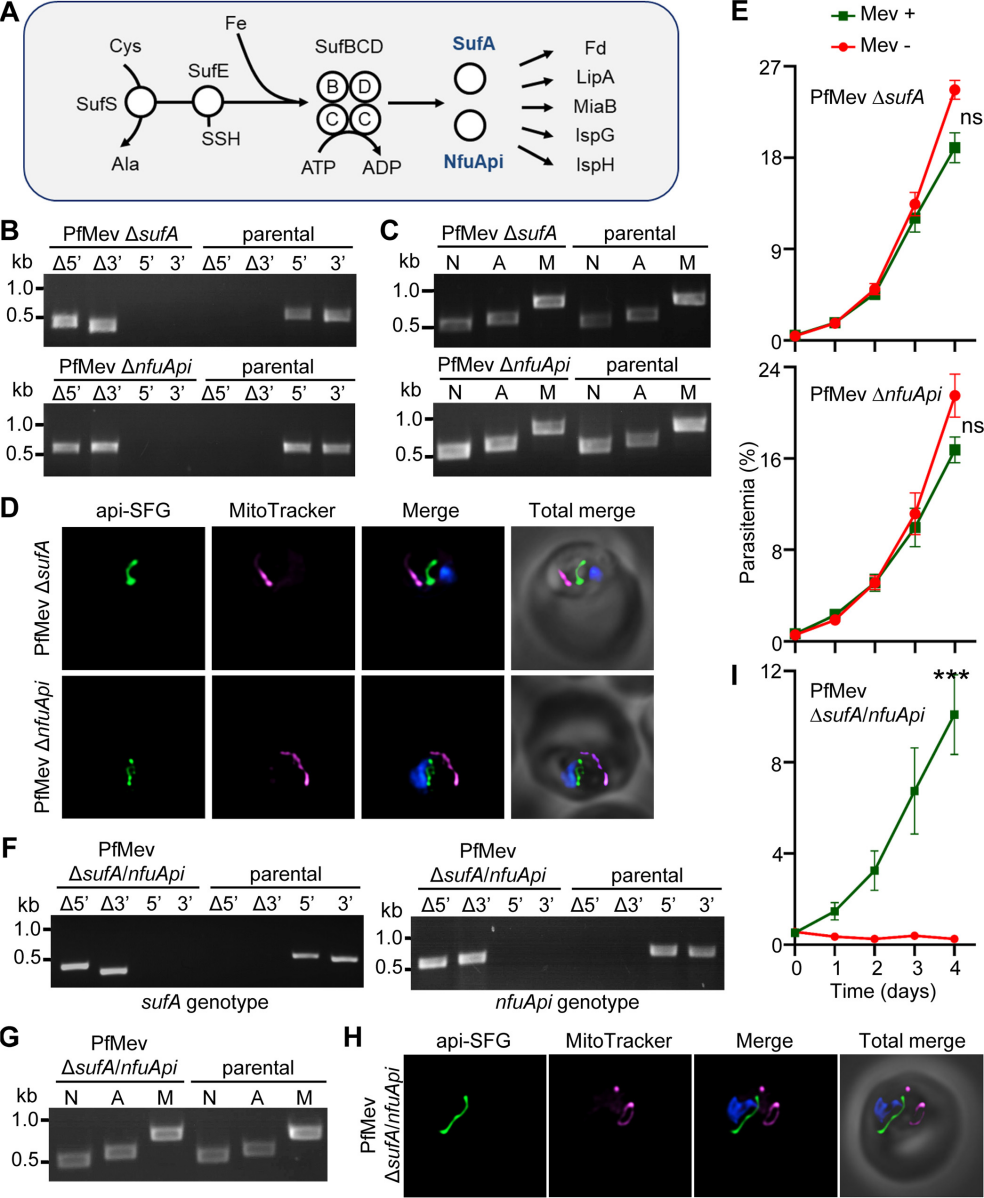
Characterization of PfMev Δ*sufA*, PfMev Δ*nfuApi*, and PfMev Δ*sufA* Δ*nfuApi* double-deletion parasite lines. (A) The SUF pathway of P. falciparum. SufA and NfuApi (in blue) receive FeS cofactors from the SufBC_2_D complex and transfer them to the downstream FeS-dependent proteins. Cys, cysteine; Ala, alanine; Fe, iron. (B) Genotyping PCR to confirm deletion of *sufA* or *nfuApi* in PfMev parasites. In both PfMev Δ*sufA* (top) and PfMev Δ*nfuApi* (bottom) parasite lines, presence of PCR products for the Δ5′ and Δ3′ loci and lack of amplification for the 5′ and 3′ loci demonstrate successful gene deletion. The same reactions were also conducted with the PfMev line (parental) as a control. Expected amplicon sizes are provided in Table S3. (C) Parasite nuclear (N), apicoplast (A), and mitochondrial (M) genome detection by PCR amplifying *ldh* (N), *sufB* (A), and *cox1* (M) genes from the PfMev Δ*sufA* (top) and PfMev Δ*nfuApi* (bottom) parasite lines, with the PfMev parasite line (parental) serving as a positive control. Successful amplification of *sufB* in the PfMev Δ*sufA* and PfMev Δ*nfuApi* parasites indicates presence of the apicoplast genome. (D) Live epifluorescence microscopy of PfMev Δ*sufA* (top) and PfMev Δ*nfuApi* (bottom) parasites. In both parasite lines, a single intact apicoplast is seen. The api-SFG protein (green) labels the apicoplast, the mitochondrion is stained with MitoTracker (magenta), and nuclear DNA is stained with DAPI (blue). All the images were taken in the absence of mevalonate. Each image depicts a field of 10 μm by 10 μm. (E) Growth curve of PfMev Δ*sufA* (top) and PfMev Δ*nfuApi* (bottom) parasites. Asynchronous parasites from each line were cultured in the presence or absence of 50 μM mevalonate (Mev). Parasitemia was determined every 24 h by flow cytometry for 4 days. Data from two independent biological replicates, each with four technical replicates, are shown. Error bars represent standard errors of the means. ns, nonsignificant (two-way ANOVA, Šidák-Bonferroni method); *P* > 0.05. (F) Genotyping PCR to confirm deletion of both *sufA* and *nfuApi* in the PfMev Δ*sufA* Δ*nfuApi* double-knockout line. Presence of the Δ5′ and Δ3′ amplicons from the PfMev Δ*sufA* Δ*nfuApi* line indicates successful deletion of both *sufA* (left) and *nfuApi* (right) genes. The PfMev line (parental) was used as a control. Expected amplicon sizes are provided in Table S3. (G) Nuclear (N), apicoplast (A), and mitochondrial (M) genome detection by amplifying *ldh* (N), *sufB* (A), and *cox1* (M) from the PfMev Δ*sufA* Δ*nfuApi* parasite line. In this parasite line, successful amplification of *sufB* demonstrates the presence of the apicoplast genome. The PfMev parasite line (parental) was used as control. (H) Live epifluorescence microscopy of PfMev Δ*sufA* Δ*nfuApi* parasites, displaying a single intact apicoplast. The api-SFG protein (green) labels the apicoplast, the mitochondrion is stained with MitoTracker (magenta), and nuclear DNA is stained with DAPI (blue). All the images were taken in the presence of mevalonate. Each image depicts a field of 10 μm by 10 μm. (I) Growth curve of PfMev Δ*sufA* Δ*nfuApi* parasites. Asynchronous parasites were cultured in the presence or absence of 50 μM mevalonate (Mev). Parasitemia was determined every 24 h via flow cytometry for 4 days. Data from two independent biological replicates, each with four technical replicates, are shown. Error bars represent standard errors of the means. ***, *P* < 0.001 (two-way ANOVA, Šidák-Bonferroni method).

To investigate this hypothesis, we attempted to delete the FeS transfer proteins SufA (gene ID PF3D7_0522700) and NfuApi (gene ID PF3D7_0921400) (7, 33) (Fig. 4A). In Escherichia coli, both SufA and NfuA (an ortholog of NfuApi) were shown to receive FeS cofactors from the SufBC_2_D FeS assembly complex (50, 51) and transfer those clusters to downstream target enzymes (51, 52). Both SufA and NfuApi were previously localized to the apicoplast (33, 53). Additionally, SufA was shown to interact with SufB of the SufBC_2_D complex and accept FeS cofactors (54), and NfuApi appears to accept and transfer FeS cofactors as well (53). To determine the roles of these proteins in P. falciparum, we targeted both the *sufA* and *nfuApi* genes for deletion in the PfMev line under continuous supplementation with mevalonate and were successful in deleting both proteins (Fig. 4B). We were able to show that neither *sufA* nor *nfuApi* (Fig. 4C and D) is individually required for apicoplast maintenance. We also showed that neither of these parasite lines relies on mevalonate supplementation for replication (Fig. 4E) or shows a significant growth defect compared to the parental line (Fig. S1D). Taken together, these results show that both proteins are individually dispensable for parasite survival.

As mentioned above, both SufA and NfuApi can accept FeS cofactors from the SufBC_2_D complex, which suggests there might be some redundancy in the activity of these proteins. To investigate this possibility, we generated a double deletion of both *sufA* and *nfuApi* (PfMev Δ*sufA* Δ*nfuApi*) under continuous supplementation of mevalonate (Fig. 4F). Surprisingly, these parasites retained an intact apicoplast organelle (Fig. 4G and H). However, in contrast to the individual deletion lines, PfMev Δ*sufA* Δ*nfuApi* parasites were dependent on mevalonate supplementation for growth (Fig. 4I) but did not show any significant growth defect in comparison with the parental line (Fig. S1D). Collectively, these findings show that while *sufA* and *nfuApi* are not individually essential for parasite survival, they display a synthetic lethal phenotype when disrupted together, which suggests a functional redundancy between these two proteins.

## DISCUSSION

**FIG 5:**
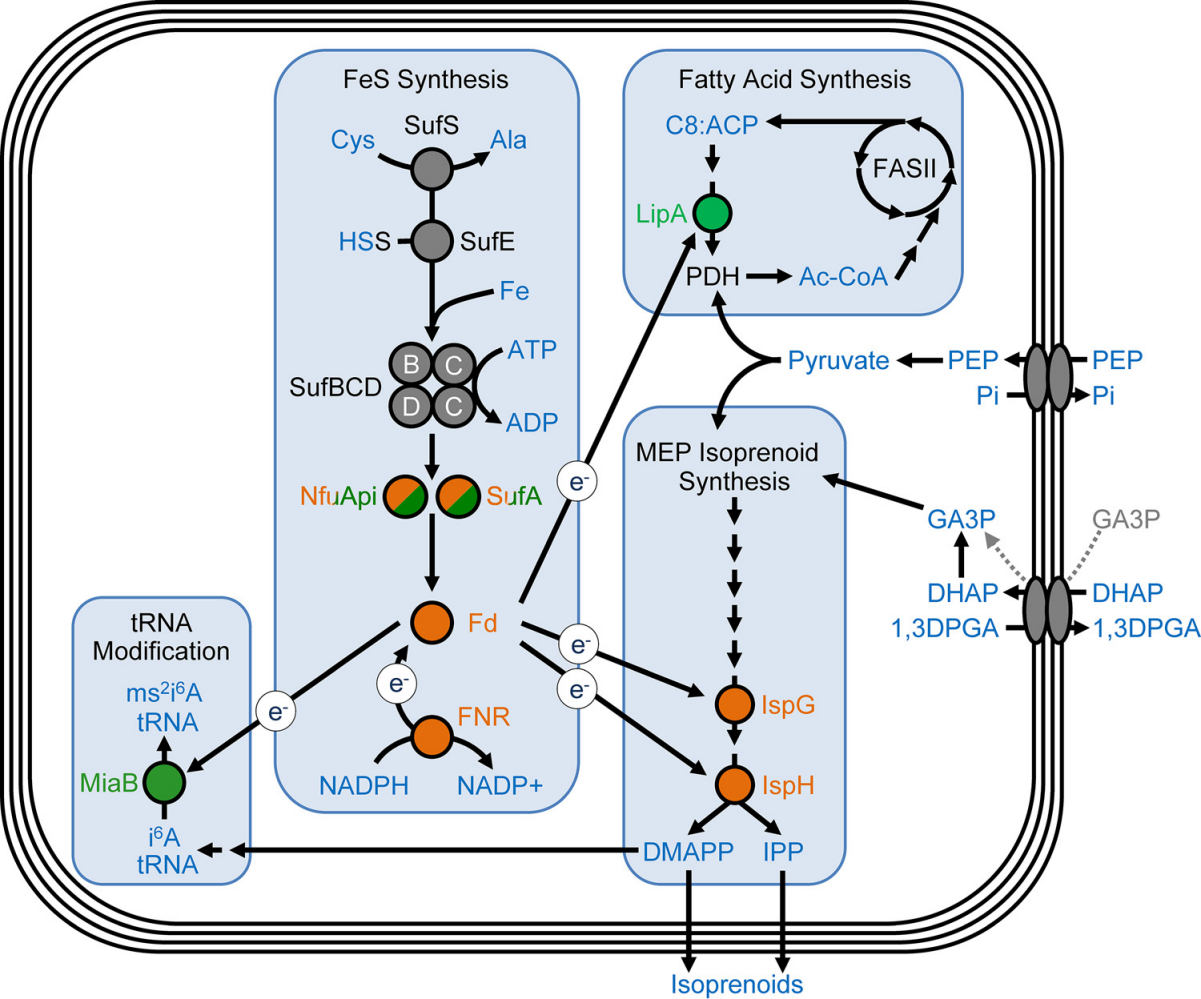
Roles of FeS-dependent proteins in the apicoplast. Simplified representation of apicoplast pathways. FeS cofactors synthesized by the SUF pathway are transferred by SufA and NfuApi to FeS-dependent proteins. Fd is reduced by the NADPH-dependent enzyme FNR and is thought to provide reducing equivalents (e^−^) to four other apicoplast enzymes. Proteins are color coded according to gene deletion results; green denotes dispensable proteins and orange denotes proteins essential for parasite survival. SufA and NfuApi are dispensable when deleted individually but are essential together, as shown by double deletion. The proteins that were not investigated in this study are in gray. Apicoplast membrane transporters are thought to import PEP and DHAP and may import GA3P directly (gray dotted arrows) (32). IspG, hydroxylmethylbutenyl diphosphate (HMB-PP) synthase; IspH, HMB-PP reductase; DMAPP, dimethylallyl pyrophosphate; IPP, isopentenyl pyrophosphate; Cys, cysteine; Ala, alanine; Fe, iron; Fd, ferredoxin; FNR, ferredoxin-NADP+ reductase; i^6^A, *N*^6^-isopentenyl adenosine; miaB, tRNA-i^6^A37 methylthiotransferase; ms^2^i^6^A, 2-methylthiol-*N*^6^-isopentenyl adenosine; C8:ACP, octanoyl acyl carrier protein; FASII, type II fatty acid synthesis pathway; LipA, lipoate synthase; PEP, phosphoenolpyruvate; GA3P, glyceraldehyde-3-phosphate; DHAP, dihydroxyacetone phosphate; 1,3 DPGA, 1,3-diphosphoglyceric acid; P_i_, inorganic phosphate.

P. falciparum Fd is an apicoplast-resident FeS-dependent protein (11), which along with FNR constitutes one of the few redox systems present within the organelle (4–6, 11–13). FNR harvests two electrons (through hydride transfer) from NADPH and uses these electrons to reduce two Fd proteins (one electron each), which in turn deliver reducing power throughout the organelle (26). Fd has been demonstrated to function as an electron donor to LipA (15) and IspH (16) and is also believed to deliver reducing power to other FeS-containing proteins, including IspG, MiaB, and proteins within the SUF biosynthesis pathway (Fig. 5) (8, 14, 17). This system is an attractive drug target because it appears to play a central role in apicoplast metabolism and is not found in the human host (9, 10).

The apicoplast appears to maintain a highly reducing environment (55). It was hypothesized that inactivation of Fd through loss of its required FeS cofactor would lead to redox dysfunction, subsequent DNA damage, and loss of the organelle (7). In E. coli, loss of the Fd/FNR system has been shown to lead to an increased sensitivity to oxidative stress (7, 28). We found that Fd and FNR are both essential for parasite survival; however, neither is required for the maintenance of the organelle (Fig. 1). Since redox homeostasis should be critical for organelle maintenance, these results suggest that other mechanisms are sufficient for maintaining redox balance and oxidative defense in the apicoplast. These additional apicoplast redox proteins include dihydrolipoyl dehydrogenase (LipDH; gene ID PF3D7_0815900) (56), glyoxalase I-like protein (GILP; gene ID PF3D7_0604700), targeted glyoxalase II (tGLO2; gene ID PF3D7_1205700) (57, 58), antioxidant protein (AOP; gene ID PF3D7_0729200), glutathione peroxidase-like thioredoxin peroxidase [TPx(GI); gene ID PF3D7_1212000], glutathione reductase (GR; gene ID PF3D7_1419800) (5), thioredoxin-like protein (ATrx2; gene ID PF3D7_0529100) (59), and lipoate-protein ligase B (LipB; gene ID PF3D7_0823600) (60). Deletion of LipB perturbed the expression of redox regulators in the apicoplast and the cytosol but did not prevent the growth of blood-stage P. falciparum parasites (60, 61). A forward genetic screen in P. falciparum indicated that the redox proteins GILP, TPx(GI), GR, and ATrx2 appear to be essential in blood-stage parasites (48). While TPx(GI), GR, and ATrx2 appear to be dually localized (5, 59), the essentiality of these redox proteins may suggest that maintaining redox balance within the organelle is a required function. A future mechanistic study might provide insights into the mechanism of redox homeostasis at play in the apicoplast in the absence of the Fd/FNR redox system.

There are several apicoplast-localized FeS proteins that rely on the Fd/FNR system (8, 15–17, 22). We generated a series of gene deletion lines targeting putative Fd-dependent proteins located in the apicoplast to determine which are essential for parasite survival. One of these Fd-dependent proteins is the 4Fe-4S cluster protein LipA (7, 19). By using yeast and bacterial two-hybrid systems, it has been shown that T. gondii LipA interacts with P. falciparum Fd, suggesting electron flow from Fd to LipA (15). LipA is involved in the lipoylation of the apicoplast-resident PDH complex (19, 41), which is the second step of lipoate synthesis. This step is preceded by the octanoyltransferase LipB, which transfers an octanoyl group from the acyl carrier protein to the E2 subunit of the PDH complex (19, 47). LipA mediates the insertion of two sulfur atoms into the octanoyl moiety of PDH, converting it to the active lipoylated form of the enzyme (19). Lipoate functions as an essential cofactor of PDH, which converts pyruvate into acetyl-CoA, which in turn is used by the FASII pathway within the apicoplast (62–64). Previous attempts to delete LipA in P. falciparum were unsuccessful (37). However, genes relevant to the activity and function of LipA, such as those encoding LipB (61), PDH subunits (32, 56, 65), and multiple components of the FASII pathway (36, 66), have been deleted in blood-stage P. falciparum parasites, suggesting that LipA should be dispensable as well. Consistent with these results, we were successful in deleting LipA and found that it is not required for the growth of blood-stage parasites (Fig. 2).

MiaB is predicted to be an apicoplast-resident tRNA-modifying enzyme which contains 4Fe-4S clusters and receives electrons from reduced Fd (21, 22, 38). MiaB acts downstream of MiaA, which is a tRNA isopentenyltransferase. MiaA uses DMAPP to isopentenylate the *N*^6^-nitrogen of adenine at base 37 (A37), forming the i^6^A37 modification (39, 67), on a subset of tRNAs that read codons beginning with uridine (Phe, Leu, Ser, Tyr, Cys, and Trp) (67, 68). Downstream of this, MiaB functions as a tRNA methylthiotransferase (19), catalyzing the methylthiolation of the i^6^A37 base, to generate the 2-methylthio-*N*^6^-isopentenyl-adenosine ms^2^i^6^A37 tRNA modification (38). In other biological systems, the ms^2^i^6^A37 tRNA modification is involved in increasing translational fidelity, reducing frameshifting, assisting in stop codon suppression, reading frame maintenance, and tRNA-ribosome binding (40–44). However, we found MiaB to be dispensable, with deletion of this gene having no discernible detrimental effect on the growth of blood-stage parasites (Fig. 2). This result suggests that the ms^2^i^6^A37 tRNA modification is not required for blood-stage parasite survival.

The other two known Fd-dependent FeS-containing proteins in the apicoplast are IspG and IspH (16, 17). These two enzymes are responsible for the last two steps of isoprenoid precursor production (16, 17, 22), with IspG predicted to catalyze the conversion of methylerythritol cyclodiphosphate (MEcPP) to hydroxylmethylbutenyl diphosphate (HMB-PP) (69), followed by IspH which converts HMB-PP into IPP and DMAPP (70). While we expected IspG and IspH to be essential due to their presumed involvement in isoprenoid precursor biosynthesis, it was also possible that deletion of these proteins could lead to loss of the apicoplast organelle. Inactivation of either enzyme could result in the toxic buildup of the substrate MEcPP, which has a critical role as a retrograde signaling metabolite in plant plastids (71, 72). Ultimately, this proved not to be the case, and while IspG and IspH are essential for parasite survival, deletion of these proteins did not result in loss of the apicoplast organelle (Fig. 3). Taken together, these results also suggest that the reduction of IspG and IspH by reduced Fd is essential for parasite survival.

Assembled FeS cofactors are delivered to the target proteins by FeS transfer proteins. In the E. coli SUF pathway, SufA can transfer both 2Fe-2S and 4Fe-4S clusters to the target proteins (51) and IscA (a SufA homolog) transfers 2Fe-2S clusters (73). Under conditions of redox stress, NfuA has been shown to transfer 4Fe-4S clusters (52). In P. falciparum, the FeS transfer proteins SufA and NfuApi should be required for metalation of Fd and all other known (and unknown) FeS proteins in the apicoplast (33, 53). We found that SufA and NfuApi are dispensable in blood-stage parasites, but a double knockout resulted in parasites that could not survive without mevalonate supplementation (Fig. 4). This is consistent with previous findings demonstrating that P. berghei parasites that lack either protein can complete the entire parasite life cycle (33, 53). Presumably, both proteins are capable of delivering clusters to all essential apicoplast FeS proteins even though apicoplast proteins are known to rely on different types of clusters; Fd contains a 2Fe-2S cluster (11), while LipA, MiaB, IspG, and IspH contain 4Fe-4S clusters (7, 14, 16–18). A similar phenomenon of functional redundancy was observed with E. coli FeS transfer proteins. Deletion of SufA or IscA is tolerated in E. coli; however, deletion of both proteins results in a severe growth defect (74). Additionally, SufA and NfuApi from P. falciparum have been expressed in E. coli and were shown to transfer FeS clusters to the same apo-protein (75), which further supports the functional redundancy of SufA and NfuApi. This double deletion of SufA and NfuApi also inactivates all downstream Fd-dependent proteins, since Fd requires FeS cofactors for activity (76).

While the gene deletions explored in this work were all conducted in blood-stage parasites, it would be informative to determine their roles and essentiality in other stages of the parasite life cycle. SufA/NfuApi together, as well as IspG, IspH, Fd, and FNR, are all essential in the blood stage and would likely be required in other parasite stages. For SufA and NfuApi, the individual dispensability at all stages in the parasite life cycle has been established in P. berghei, which may or may not extend to P. falciparum (33, 53). The essentiality of LipA has not been explored in other stages, but LipB, which along with LipA is responsible for the lipoylation of pyruvate dehydrogenase (PDH) (19), has been shown to be essential for sporogony in *Anopheles* mosquitoes (60). MiaB is dispensable in blood-stage parasites, and while there is no information on the essentiality of this or other apicoplast-resident tRNA-modifying proteins in any stages of the parasite life cycle, this would be an interesting area to explore in future studies.

When trying to understand the impact of these gene deletions on the parasite, we can also look to recent unpublished studies by Okada and coworkers (77), which demonstrated that isoprenoid biosynthesis is required for normal apicoplast morphology. Inhibition of this pathway with fosmidomycin, which targets deoxyxylulose-5-phosphate reductoisomerase (DXR), or removal of mevalonate in PfMev Δ*dxs* parasites (deletion of deoxyxylulose-5-phosphate synthase) prevents normal apicoplast elongation and branching but does not cause organelle disruption. The same results would likely be seen with the essential genes studied in this work. IspG and IspH are also involved in isoprenoid biosynthesis. Additionally, since IspG and IspH contain FeS clusters that are critical for their enzymatic activity, Fd, FNR, and SufA/NfuApi, which are involved in FeS transfer, would also likely yield these results as well.

Through the work described here, we determined that Fd, FNR, IspG, and IspH have essential roles in the survival of blood-stage malaria parasites but are not required for maintenance of the apicoplast. Although LipA and MiaB have been hypothesized to be essential proteins, both can be deleted without any significant growth phenotype. Our results are consistent with a model in which the MEP pathway proteins IspG and IspH cannot function without electron transfer from the Fd/FNR system (Fig. 5). A recent study in T. gondii also supports our model (78). Since the essential FeS proteins contain 2Fe-2S and 4Fe-4S clusters, both cluster types are needed. We found that either SufA or NfuApi can accomplish FeS transfer, demonstrating functional redundancy in the transfer of both cluster types. Overall, the work outlined here provides insights into the apicoplast-resident Fd/FNR system and the roles of the proteins upstream and downstream of this redox system.

## MATERIALS AND METHODS

### P. falciparum culture and maintenance.

Unless otherwise noted, blood-stage P. falciparum parasites were cultured in human erythrocytes at 1% hematocrit in a 10-mL volume of CMA (complete medium with Albumax) containing RPMI 1640 medium with l-glutamine (United States Biological Life Sciences), supplemented with 20 mM HEPES, 0.2% sodium bicarbonate, 12.5 μg/mL hypoxanthine, 5 g/L Albumax II (Life Technologies), and 25 μg/mL gentamicin. Cultures were maintained in 25-cm^2^ gassed flasks (94% N_2_, 3% O_2_, 3% CO_2_) and incubated at 37°C.

### Generation of P. falciparum plasmid constructs for gene deletion.

Target genes were deleted using previously published Cas9-mediated gene editing methods (31). The homology repair plasmid pL8 (34) or pRS (31) was used in combination with the Cas9-expressing pUF1-Cas9 plasmid (34). Alternatively, genes were targeted for deletion using the pRSng (32) or pRSng (BSD) plasmid in combination with the pCasG plasmid (31). To generate the pRSng (BSD) plasmid, harmonized blasticidin-*S*-deaminase (hBSD) was cut with BamHI and HindIII from a synthetic plasmid (79) and ligated into the same sites in the pRSng plasmid to replace the sequence encoding human dihydrofolate reductase (hDHFR).

For generation of the deletion constructs, homology arms of ∼300 to 600 bp were amplified from P. falciparum strain NF54 genomic DNA (gDNA) using the homology arm 1 (HA1) and HA2 forward and reverse primers corresponding to each gene (Table S1) and inserted into the repair plasmids using ligation-independent cloning (LIC) methods (In-Fusion; Clontech). The repair plasmids were digested with NotI for insertion of HA1 and with NgoMIV for insertion of HA2. Guide RNA sequences were synthesized as oligonucleotides (Table S1), annealed, and inserted using LIC.

### P. falciparum transfections for gene deletion.

The PfMev parental line (31) was used for all gene deletion experiments. This line expresses apicoplast-trafficked Super Folder Green (api-SFG) (35) and four enzymes that produce the isoprenoid precursors IPP and DMAPP from mevalonate. Transfections to generate single-gene-knockout lines were conducted as previously described (80) with the corresponding plasmids (Table S2). Briefly, 400 μL of red blood cells (RBCs) were electroporated with 75 μg each of the Cas9 expression plasmid and the corresponding homology repair plasmid. The transfected RBCs were mixed with synchronized schizont-stage PfMev parasites and maintained in 10 mL CMA containing 50 μM mevalonate (racemic mevalonolactone; Sigma-Aldrich). After ∼48 h, 1.5 μM 5-methyl[1,2,4]triazolo[1,5-a]pyrimidin-7-yl naphthalen-2-ylamine (DSM1; BEI Resources) and 2.5 nM WR99210 (Jacobus Pharmaceuticals) were added to ensure the retention of both transfection plasmids, in the presence of 50 μM mevalonate. After 7 days of drug selection, the parasites were switched to medium containing only 50 μM mevalonate. Infected RBCs were first observed 17 to 25 days after beginning drug selection. Once parasites were observed, 2.5 nM WR99210 along with 50 μM mevalonate was reintroduced into the growth medium to maintain the integrated hDHFR drug resistance cassette.

10.1128/mBio.03023-21.3 TABLE S2 Plasmids used for generation of gene deletion lines. Download Table S2, DOCX file, 0.02 MB. Copyright © 2022 Swift et al. 2022 Swift et al. https://creativecommons.org/licenses/by/4.0/ This content is distributed under the terms of the Creative Commons Attribution 4.0 International license.

To generate the PfMev Δ*sufA* Δ*nfuApi* double knockout, RBCs were transfected with the pCasG-*nfuApi* and pRSng (BSD)-*nfuApi* repair plasmids as described above. The transfected RBCs were mixed with synchronized PfMev Δ*sufA* parasites and cultured in 10 mL medium with 50 μM mevalonate. Blasticidin (2.5 μg/mL; Corning Inc.), 1.5 μM DSM1, and 2.5 nM WR99210 were added 48 h posttransfection to the culture medium containing 50 μM mevalonate. After a 7-day selection period, the culture was switched to complete medium containing 2.5 nM WR99210 and 50 μM mevalonate until parasites appeared. Upon parasite appearance, 2.5 μg/mL blasticidin was reintroduced into the growth medium with WR99210 and mevalonate. Independent lines were obtained for each gene deletion from a minimum of two independent transfections (Table S2). All the subsequent analysis described in this study on all the deletion lines were conducted ∼60 to 90 days posttransfection.

### Confirmation of gene deletions.

Primers were designed to screen for 5′ integration (Δ5′ reaction primers GOI.5.F and pRS.R) and 3′ integration (Δ3′ reaction primers pRS.F and GOI.3.R) of the gene disruption cassette and the wild-type (WT) 5′ region (primers GOI.5.F and GOI.5.WT.R) and WT 3′ region (primers GOI.3.WT.F and GOI.3.R) of the gene of interest (GOI) (Table S1). The parental PfMev line was used as a control for these reactions. For the PCRs, 1 μL of parasite culture was added to a 50-μL PCR volume. The expected PCR amplicon sizes for the confirmation PCRs are provided in Table S3.

### Confirmation of apicoplast loss.

The presence of the apicoplast organellar genome was detected by PCR with primers (SufB.F and SufB.R) specific for the *sufB* gene (gene ID PF3D7_API04700). Control PCRs with LDH.F and LDH.R primers amplified the lactate dehydrogenase (ldh; gene ID PF3D7_1324900) gene from the nuclear genome and Cox1.F and Cox1.R primers amplified the cytochrome *c* oxidase subunit 1 (*cox1* and mal_mito_2) from the mitochondrial genome (Table S1). For the PCRs, 1 μL of parasite culture was added to a 50-μL PCR volume. The parental PfMev line was used as a positive control for apicoplast genome detection. The expected amplicon sizes for *ldh*, *sufB*, and *cox1* are 520 bp, 581 bp, and 761 bp, respectively.

### Live-cell epifluorescence microscopy.

Approximately 100 μL of parasite culture was incubated with 30 nM MitoTracker CMX-Ros (Invitrogen) and 1 μg/mL 4′,6-diamidino-2-phenylindole (DAPI; Invitrogen) for 30 min at 37°C. Cells were then washed three times with 100 μL of CMA and incubated for 5 min at 37°C after each wash. Cells were resuspended in 20 μL of CMA (with or without 50 μM mevalonate; see figure legends for details) and then pipetted onto slides and sealed with wax for observation on a Zeiss AxioImager M2 microscope (Carl Zeiss Microscopy, LLC) equipped with a Hamamatsu ORCA-R2 camera (Hamamatsu Photonics) using a 100×/1.4 numerical aperture (NA) lens. A series of images spanning 5 μm in the z-plane were acquired with 0.2-μm spacing, and images were deconvolved with VOLOCITY software (PerkinElmer) to report a single image in the z-plane.

### Testing mevalonate dependence in gene-deletion PfMev lines via flow cytometry.

Gene deletions generated in the PfMev line were tested to determine reliance on mevalonate for survival. Parasite lines were washed with 10 mL CMA three times to remove any mevalonate from the culture medium and then cultured in CMA alone or CMA with 50 μM mevalonate. Asynchronous parasites were seeded in a 96-well plate (Corning Inc.) at a 0.5% starting parasitemia and 2% hematocrit in a total volume of 250 μL, in quadruplicate for each condition. Plates were incubated in chambers gassed with 94% N_2_, 3% O_2_, and 3% CO_2_ at 37°C. Parasite samples were collected, and the culture medium was exchanged daily for 4 days.

For growth curve determination, parasites were stained with SYBR green (Invitrogen) and analyzed via flow cytometry. Parasitemia was counted on the same day as seeding, after which parasites were collected every 24 h. Samples collected on days 1 to 3 were diluted 1:10 in phosphate-buffered saline (PBS) and stored in a 96-well plate at 4°C. On day 4, parasites were stained with SYBR green by transferring 1 μL of parasite culture, or 10 μL of the 1:10 dilutions, to a 96-well plate containing 100 μL of 1× SYBR green (Invitrogen) per well in PBS. Plates were incubated at room temperature for 30 min while shaking, under protection from light. Postincubation, 150 μL of PBS was added to each well to dilute unbound SYBR green dye. Samples were analyzed with an Attune Nxt flow cytometer (Thermo Fisher Scientific), with a 50-μL acquisition volume and a running speed of 25 μL/min, with 10,000 total events collected. All growth assays presented in this work were conducted in two biological replicates, each with quadruplicate technical replicates.

### Determining fitness cost of gene deletion lines.

The growth rates of the parental and gene knockout lines were compared to determine whether the gene deletions affected parasite growth. To determine growth rate for each line, asynchronous parasites were seeded at 0.5% initial parasitemia and 2% hematocrit in a 96-well plate in a total volume of 250 μL, in duplicate. After 48 h (a complete growth cycle), final parasitemia was determined using flow cytometry, and growth rate was calculated by dividing the final parasitemia by the initial parasitemia. For each parasite line, we repeated this experiment for at least five growth cycles. The growth rate data are presented as fold increase per growth cycle. For flow cytometry, parasites were stained and analyzed as described above.
