# Supplementary material for: Roles of Ferredoxin-Dependent Proteins in the Apicoplast of Plasmodium falciparum Parasites
Source: mBio. 2022 Feb 15;13(1):e03023-21. doi: 10.1128/mbio.03023-21 (PMC8844926; doi:10.1128/mbio.03023-21)
Supplement: TABLE S1 [file mbio.03023-21-st001.docx]

**Table S1.** **Primers and oligonucleotide used in this study.**

| Primer Name | Primer Sequence |
| --- | --- |
| MiaB.HA1.F | GCCACGAGCGGCCTTAGCAGGAGGAGGAACGTATTAC |
| MiaB.HA1.R | AAGCGCAGCGGCCTAAATGGGTCCATGTTATTATCTGTAG |
| MiaB.HA2.F | CGACAGACGCCGGTTTTTCTGATATAGAAAATGAATATTG |
| MiaB.HA2.R | GGCCACCAGCCGGTACGCACCCACATACAATAATTTTTAT |
| Fd.HA1.F | GCCACGAGCGGCCACCAAAAAGGCTATTGCATCAATAAAC |
| Fd.HA1.R | AAGCGCAGCGGCCTCAAAAATAATTTACTCTTTTGGCTTC |
| Fd.HA2.F | CGACAGACGCCGGTAGTTGTTCTACATGCGCAGCAAAAT |
| Fd.HA2.R | GGCCACCAGCCGGAGGAAATTTTATCATTCCCCATTTCAA |
| FNR.HA1.F | GCCACGAGCGGCCTGAAAATTCGTTTCGTCTTTATCTTGT |
| FNR.HA1.R | AAGCGCAGCGGCCTTCCAAATATTTAAAGAGACCAT |
| FNR.HA2.F | CGACAGACGCCGGTGCACATGGATATTTTAATTTACCA |
| FNR.HA2.R | GGCCACCAGCCGGTCATGACTTTTCAAAATATCCATAAC |
| LipA.HA1.F | GTGCCACGAGCGGCCCCCATCCCTTTTTTACTTGTATATC |
| LipA.HA1.R | TTAAGCGCAGCGGCCCAGCTACATGAAACCAGTCTG |
| LipA.HA2.F | TTCGACAGACGCCGGGCTAGTCATTTTGCTAAAACAGTAG |
| LipA.HA2.R | ATGGCCACCAGCCGGCGAACTAAAGGGCCGCTG |
| IspH.HA1.F | GTGCCACGAGCGGCCATGTCAGTTACCACATTTTGTTC |
| IspH.HA1.R | TTAAGCGCAGCGGCCCTTATCTGTTCTTGTTTTCCGAAG |
| IspH.HA2.F | TCGACAGACGCCGGGATTGTGCACTTATCGTACAAAAACT |
| IspH.HA2.R | ATGGCCACCAGCCGGTGCAAGAGATTCTTGGGAAGC |
| IspG.HA1.F | GTGCCACGAGCGGCCTCCCAACACGAGAAGTAGTTATTG |
| IspG.HA1.R | TTAAGCGCAGCGGCCCTACCATACCTAATGGTGTATCTCC |
| IspG.HA2.F | TTCGACAGACGCCGGGCAGGAAGTTGTTTGATGGAT |
| IspG.HA2.R | TGGCCACCAGCCGGCAATTTATCACAAGCTTCTTCCTCAG |
| SufA.HA1.F | GCCACGAGCGGCCATAATATGCTTTTACGCATATTTGAAG |
| SufA.HA1.R | AAGCGCAGCGGCCAGAAAACACAAAAAAATGTGTATTGTC |
| SufA.HA2.F | CGACAGACGCCGGTGGAGGATTTAAGTAAAAAAAACCTTA |
| SufA.HA2.R | GGCCACCAGCCGGTCATATATCACACATTGAATGATTTTC |
| NfuApi.HA1.F | GCCACGAGCGGCCGAACGGTTCAGGTAAAATTTAGAAAAA |
| NfuApi.HA1.R | AAGCGCAGCGGCCCTAAGGTTTTTCACATACACAAAAAAA |
| NfuApi.HA2.F | CGACAGACGCCGGGTTCCCTACATTAACTGTTAATTTTGA |
| NfuApi.HA2.R | GGCCACCAGCCGGTCCTTTTATTTATTTGTTTGTTTGTTT |
| MiaB.gRNA.F | TAAGTATATAATATTTGCTTATCAACATCTTCCTCGTTTTAGAGCTAGAA |
| MiaB.gRNA.R | TTCTAGCTCTAAAACGAGGAAGATGTTGATAAGCAAATATTATATACTTA |
| Fd.gRNA.F | TAAGTATATAATATTTTACCATATAGTTGTAGGGGGTTTTAGAGCTAGAA |
| Fd.gRNA.R | TTCTAGCTCTAAAACCCCCTACAACTATATGGTAAAATATTATATACTTA |
| FNR.gRNA.F | TAAGTATATAATATTTATTTGGAAGGCCATACTTGGTTTTAGAGCTAGAA |
| FNR.gRNA.R | TTCTAGCTCTAAAACCAAGTATGGCCTTCCAAATAAATATTATATACTTA |
| LipA.gRNA.F | TAAGTATATAATATTAGAGATGATTTACCAGATGGGTTTTAGAGCTAGAA |
| LipA.gRNA.R | TTCTAGCTCTAAAACCCATCTGGTAAATCATCTCTAATATTATATACTTA |
| IspH.gRNA.F | TAAGTATATAATATTTACACAAACCACACTAAGTAGTTTTAGAGCTAGAA |
| IspH.gRNA.R | TTCTAGCTCTAAAACTACTTAGTGTGGTTTGTGTAAATATTATATACTTA |
| IspG.gRNA.F | TAAGTATATAATATTACCATTAGGTATGGTAGAATGTTTTAGAGCTAGAA |
| IspG.gRNA.R | TTCTAGCTCTAAAACATTCTACCATACCTAATGGTAATATTATATACTTA |
| SufA.gRNA.F | TAAGTATATAATATTAGTAATGATTTAATTAGTGGGTTTTAGAGCTAGAA |
| SufA.gRNA.R | TTCTAGCTCTAAAACCCACTAATTAAATCATTACTAATATTATATACTTA |
| NfuApi.gRNA.F | TAAGTATATAATATTTGTCATATTGTAAGATGCTAGTTTTAGAGCTAGAA |
| NfuApi.gRNA.R | TTCTAGCTCTAAAACTAGCATCTTACAATATGACAAATATTATATACTTA |
| pRS.R . | TACAAAATGCTTAAGCGCAGCGGCC |
| pRS.F | CATATTTATTAAATCTAGAATTCGACAGACGCCG |
| MiaB.5.F | GATATTAAATGGGAAAGCAAAACTTATAAGTTTTTTATTC |
| MiaB.3.R | GTGATACATTTTCAAATATCTCCATATCTTTTTCTG |
| MiaB.5.WT.R | GATGTTGATAAGCATTCCAAGTCATATTCTC |
| MiaB.3.WT.F | GGATGTGCTCATAATAGTTCAGATTCTG |
| Fd.5.F | CTGGTATATTATAGTTTATATTTAATAAGAAAAGGGC |
| Fd.3.R | GGTAGCGAAGTTAATACATACACATATATATATGTG |
| Fd.5.WT.R | CTAGCATCTAATATATATTCATCTTCATTACATTCG |
| Fd.3.WT.F | CCTAAGTAATAATCAGCTAGCTAATTCTAATAAAC |
| FNR.5.F | GAGAAGTTTTCTTTATATCATCCTATTTTTTTTGTCATAT |
| FNR.3.R | AACTAGTACACCTCAACGTGCACCCTT |
| FNR.5.WT.R | CCTGAACAATAACCATAATTTGTAATATTGGGAG |
| FNR.3.WT.F | GTGGTATTATACCTTACTATAATGAACTTGATAATAATCC |
| LipA.5.F | CCTACATAAACTGTAATGTTTCAATATGACC |
| LipA.3.R | GGTTAACTAAATTTTTTATAAAATATTCACCTGC |
| LipA.5.WT.R | GTGCTTCTTCACATACGGTATGTAAG |
| LipA.3.WT.F | CTTTCAATACAGCTAAAGCTATATGTGAATG |
| IspH.5.F | CCAGTAAGCAAAATATATCCATTCTGTATAC |
| IspH.3.R | TGTGATTTCATTTTTCTCTTTCTTTTATCA |
| IspH.5.WT.R | GGGTACATATATATTTTTCGATTCCCATC |
| IspH.3.WT.F | CCATTAAATAAAAAGTTATTCTATGTTACACAAACC |
| IspG.5.F | CTAAATGAAGAAGGGAATTCTTCAAAAAAG |
| IspG.3.R | CATATTCAATTTATGGATCTTTCCATTTG |
| IspG.5.WT.R | CAATACATAAATCAGAAAACTCAAAAGCC |
| IspG.3.WT.F | GTATTAATAACCAGAAGAATTAATGAACTTTTACAATC |
| SufA.5.F | CTTTTTTCTCCTTTTAATATTAAAAAGAAAAAAG |
| SufA.3.R | CCTAAAGATATATATTTATGTGTTATCTTATATATTTTTG |
| SufA.5.WT.R | GCTCTTATTGTTATATAAAAATGTTTTTGGAG |
| SufA.3.WT.F | GATATAGAACGAAATTGAAGAAGATGATTATATAC |
| NfuApi.5.F | ATACGAAATGAAATTAGTTTTTTAATAATATATTTATACC |
| NfuApi.3.R | CGTTAATGTGGAGAAAATTCAAAGACCTAC |
| NfuApi.5.WT.R | CGAAATTATTTTGTATAAATAACACCTTTGC |
| NfuApi.3.WT.F | CACACAACGTTAAAAATGAAATAACAGAAAG |
| LDH.F | GGAGATGTAGTTTTGTTCGATATTG |
| LDH.R | CTTGTAAAGGGATACCACCTACAG |
| SufB.F | CATGTAGCTATAGTAGAAATAATAGTAAAAGATTATGG |
| SufB.R | GACTCTGAAATACTTAAACCACGTTGC |
| Cox1.F | CTTCATCTTTAAGAATAATTGCACAAGAAAATGTAAATC |
| Cox1.R | GTACATATGATGTACCCATACTAAGCTTCC |
