# Supplementary material for: Roles of Ferredoxin-Dependent Proteins in the Apicoplast of Plasmodium falciparum Parasites
Source: mBio. 2022 Feb 15;13(1):e03023-21. doi: 10.1128/mbio.03023-21 (PMC8844926; doi:10.1128/mbio.03023-21)
Supplement: TABLE S3 [file mbio.03023-21-st003.docx]

**Table S3.** **Primer combinations and expected amplicon sizes for genotype validation PCR reactions. bp, base pair.**

|  | **PfMev ∆*fd* line (Figure 1B, top)** | | | | |
| --- | --- | --- | --- | --- | --- |
| Lane | PCR Reaction | Primer Combination | | Amplicon Size (bp) | Parasite Line |
| 1 | ∆5’ | Fd.5.F | pRS.R | 438 | PfMev ∆*fd* |
| 2 | ∆3’ | pRS.F | Fd.3.R | 568 |  |
| 3 | 5’ | Fd.5.F | Fd.5.WT.R | - |  |
| 4 | 3’ | Fd.3.WT.F | Fd.3.R | - |  |
| 5 | ∆5’ | Fd.5.F | pRS.R | - | PfMev parental line |
| 6 | ∆3’ | pRS.F | Fd.3.R | - |  |
| 7 | 5’ | Fd.5.F | Fd.5.WT.R | 636 |  |
| 8 | 3’ | Fd.3.WT.F | Fd.3.R | 794 |  |

|  | **PfMev ∆*fnr* line (Figure 1B, bottom)** | | | | |
| --- | --- | --- | --- | --- | --- |
| Lane | PCR Reaction | Primer Combination | | Amplicon Size (bp) | Parasite Line |
| 1 | ∆5’ | FNR.5.F | pRS.R | 514 | PfMev ∆*fnr* |
| 2 | ∆3’ | pRS.F | FNR.3.R | 524 |  |
| 3 | 5’ | FNR.5.F | FNR.5.WT.R | - |  |
| 4 | 3’ | FNR.3.WT.F | FNR.3.R | - |  |
| 5 | ∆5’ | FNR.5.F | pRS. R | - | PfMev parental line |
| 6 | ∆3’ | pRS. F | FNR.3.R | - |  |
| 7 | 5’ | FNR.5.F | FNR.5.WT.R | 758 |  |
| 8 | 3’ | FNR.3.WT.F | FNR.3.R | 793 |  |

|  | **PfMev ∆*lipA* line (Figure 2B)** | | | | |
| --- | --- | --- | --- | --- | --- |
| Lane | PCR Reaction | Primer Combination | | Amplicon Size (bp) | Parasite Line |
| 1 | ∆5’ | LipA.5.F | pRS.R | 639 | PfMev ∆*lipA* |
| 2 | ∆3’ | pRS.F | LipA.3.R | 567 |  |
| 3 | 5’ | LipA.5.F | LipA.5.WT.R | - |  |
| 4 | 3’ | LipA.3.WT.F | LipA.3.R | - |  |
| 5 | ∆5’ | LipA.5.F | pRS.R | - | PfMev parental line |
| 6 | ∆3’ | pRS.F | LipA.3.R | - |  |
| 7 | 5’ | LipA.5.F | LipA.5.WT.R | 692 |  |
| 8 | 3’ | LipA.3.WT.F | LipA.3.R | 618 |  |

|  | **PfMev ∆*miaB* line (Figure 2G)** | | | | |
| --- | --- | --- | --- | --- | --- |
| Lane | PCR Reaction | Primer Combination | | Amplicon Size (bp) | Parasite Line |
| 1 | ∆5’ | MiaB.5.F | pRS.R | 717 | PfMev ∆*miaB* |
| 2 | ∆3’ | pRS.F | MiaB.3.R | 579 |  |
| 3 | 5’ | MiaB.5.F | MiaB.5.WT.R | - |  |
| 4 | 3’ | MiaB.3.WT.F | MiaB.3.R | - |  |
| 5 | ∆5’ | MiaB.5.F | pRS.R | - | PfMev parental line |
| 6 | ∆3’ | pRS.F | MiaB.3.R | - |  |
| 7 | 5’ | MiaB.5.F | MiaB.5.WT.R | 1005 |  |
| 8 | 3’ | MiaB.3.WT.F | MiaB.3.R | 808 |  |

|  | **PfMev ∆*ispG* line (Figure 3B, top)** | | | | |
| --- | --- | --- | --- | --- | --- |
| Lane | PCR Reaction | Primer Combination | | Amplicon Size (bp) | Parasite Line |
| 1 | ∆5’ | IspG.5.F | pRS.R | 667 | PfMev ∆*ispG* |
| 2 | ∆3’ | pRS.F | IspG.3.R | 563 |  |
| 3 | 5’ | IspG.5.F | IspG.5.WT.R | - |  |
| 4 | 3’ | IspG.3.WT.F | IspG.3.R | - |  |
| 5 | ∆5’ | IspG.5.F | pRS.R | - | PfMev parental line |
| 6 | ∆3’ | pRS.F | IspG.3.R | - |  |
| 7 | 5’ | IspG.5.F | IspG.5.WT.R | 676 |  |
| 8 | 3’ | IspG.3.WT.F | IspG.3.R | 652 |  |

|  | **PfMev ∆*ispH* line (Figure 3B, bottom)** | | | | |
| --- | --- | --- | --- | --- | --- |
| Lane | PCR Reaction | Primer Combination | | Amplicon Size (bp) | Parasite Line |
| 1 | ∆5’ | IspH.5.F | pRS.R | 726 | PfMev ∆*ispH* |
| 2 | ∆3’ | pRS.F | IspH.3.R | 493 |  |
| 3 | 5’ | IspH.5.F | IspH.5.WT.R | - |  |
| 4 | 3’ | IspH.3.WT.F | IspH.3.R | - |  |
| 5 | ∆5’ | IspH.5.F | pRS.R | - | PfMev parental line |
| 6 | ∆3’ | pRS.F | IspH.3.R | - |  |
| 7 | 5’ | IspH.5.F | IspH.5.WT.R | 777 |  |
| 8 | 3’ | IspH.3.WT.F | IspH.3.R | 502 |  |

|  | **PfMev ∆*sufA* line (Figure 4B, top)** | | | | |
| --- | --- | --- | --- | --- | --- |
| Lane | PCR Reaction | Primer Combination | | Amplicon Size (bp) | Parasite Line |
| 1 | ∆5’ | SufA.5.F | pRS.R | 387 | PfMev ∆*sufA* |
| 2 | ∆3’ | pRS.F | SufA.3.R | 383 |  |
| 3 | 5’ | SufA.5.F | SufA.5.WT.R | - |  |
| 4 | 3’ | SufA.3.WT.F | SufA.3.R | - |  |
| 5 | ∆5’ | SufA.5.F | pRS.R | - | PfMev parental line |
| 6 | ∆3’ | pRS.F | SufA.3.R | - |  |
| 7 | 5’ | SufA.5.F | SufA.5.WT.R | 484 |  |
| 8 | 3’ | SufA.3.WT.F | SufA.3.R | 449 |  |

|  | **PfMev ∆*nfuApi* line (Figure 4B, bottom)** | | | | |
| --- | --- | --- | --- | --- | --- |
| Lane | PCR Reaction | Primer Combination | | Amplicon Size (bp) | Parasite Line |
| 1 | ∆5’ | NfuApi.5.F | pRS.R | 541 | PfMev  ∆*nfuApi* |
| 2 | ∆3’ | pRS.F | NfuApi.3.R | 557 |  |
| 3 | 5’ | NfuApi.5.F | NfuApi.5.WT.R | - |  |
| 4 | 3’ | NfuApi.3.WT.F | NfuApi.3.R | - |  |
| 5 | ∆5’ | NfuApi.5.F | pRS.R | - | PfMev parental line |
| 6 | ∆3’ | pRS.F | NfuApi.3.R | - |  |
| 7 | 5’ | NfuApi.5.F | NfuApi.5.WT.R | 566 |  |
| 8 | 3’ | NfuApi.3.WT.F | NfuApi.3.R | 565 |  |

|  | **PfMev ∆*sufA/nfuApi* line (Figure 4F, left)** | | | | |
| --- | --- | --- | --- | --- | --- |
| Lane | PCR Reaction | Primer Combination | | Amplicon Size (bp) | Parasite Line |
| 1 | ∆5’ | SufA.5.F | pRS.R | 387 | PfMev ∆*sufA/nfuApi* |
| 2 | ∆3’ | pRS.F | SufA.3.R | 383 |  |
| 3 | 5’ | SufA.5.F | SufA.5.WT.R | - |  |
| 4 | 3’ | SufA.3.WT.F | SufA.3.R | - |  |
| 5 | ∆5’ | SufA.5.F | pRS.R | - | PfMev parental line |
| 6 | ∆3’ | pRS.F | SufA.3.R | - |  |
| 7 | 5’ | SufA.5.F | SufA.5.WT.R | 484 |  |
| 8 | 3’ | SufA.3.WT.F | SufA.3.R | 449 |  |

|  | **PfMev ∆*sufA/nfuApi* line (Figure 4F, right)** | | | | |
| --- | --- | --- | --- | --- | --- |
| Lane | PCR Reaction | Primer Combination | | Amplicon Size (bp) | Parasite Line |
| 1 | ∆5’ | NfuApi.5.F | pRS. R | 541 | PfMev ∆*sufA/nfuApi* |
| 2 | ∆3’ | pRS. F | NfuApi.3.R | 557 |  |
| 3 | 5’ | NfuApi.5.F | NfuApi.5.WT.R | - |  |
| 4 | 3’ | NfuApi.3.WT.F | NfuApi.3.R | - |  |
| 5 | ∆5’ | NfuApi.5.F | pRS. R | - | PfMev parental line |
| 6 | ∆3’ | pRS. F | NfuApi.3.R | - |  |
| 7 | 5’ | NfuApi.5.F | NfuApi.5.WT.R | 566 |  |
| 8 | 3’ | NfuApi.3.WT.F | NfuApi.3.R | 565 |  |
