# Supplementary material for: Roles of Ferredoxin-Dependent Proteins in the Apicoplast of Plasmodium falciparum Parasites
Source: mBio. 2022 Feb 15;13(1):e03023-21. doi: 10.1128/mbio.03023-21 (PMC8844926; doi:10.1128/mbio.03023-21)
Supplement: TABLE S2 [file mbio.03023-21-st002.docx]

**Table S2. Plasmids used for generation of gene deletion lines.**

| Gene deletion lines | Target gene | Cas9 plasmid | Repair plasmid | No. of attempted transfections | No. of successful transfections |
| --- | --- | --- | --- | --- | --- |
| PfMev ∆*fd* | *fd* | pUF1-Cas9 | pL8-*fd* | 7 | 4 |
| PfMev ∆*fnr* | *fnr* | pUF1-Cas9 | pL8-*fnr* | 2 | 2 |
| PfMev ∆*ispG* | *ispG* | pUF1-Cas9 | pL8-*ispG* | 3 | 3 |
| PfMev ∆*ispH* | *ispH* | pUF1-Cas9 | pL8-*ispH* | 2 | 2 |
| PfMev ∆*lipA* | *lipA* | pUF1-Cas9 | pL8-*lipA* | 4 | 3 |
| PfMev ∆*miaB* | *miaB* | pUF1-Cas9 | pL8-*miaB* | 7 | 7 |
| PfMev ∆*sufA* | *sufA* | pCasG-*sufA* | pRSng-*sufA* | 8 | 6 |
| PfMev ∆*nfuApi* | *nfuApi* | pCasG-*nfuApi* | pRSng-*nfuApi* | 5 | 5 |
| PfMev ∆*sufA/nfuApi* | *sufA*  *nfuApi* | pCasG-*sufA*  pCasG-*nfuApi* | pRSng(BSD)-*sufA*  pRSng-*nfuApi* | 3 | 3 |
